# Association of Habitually Low Intake of Dietary Calcium with Blood Pressure and Hypertension in a Population with Predominantly Plant-Based Diets

**DOI:** 10.3390/nu10050603

**Published:** 2018-05-12

**Authors:** Ziqi Liu, Aiping Fang, Jingjing He, Xin Shen, Rong Gao, Xintian Zhao, Keji Li

**Affiliations:** 1 Department of Nutrition and Food Hygiene, School of Public Health, Peking University; Beijing 100191, China; liuziqi@bjmu.edu.cn (Z.L.); fangaip@mail.sysu.edu.cn (A.F.); hejingjing89@bjmu.edu.cn (J.H.); xinsh@bjmu.edu.cn (X.S.); gaorong4121058@163.com (R.G.); xintianzhao@126.com (X.Z.); 2 Department of Nutrition, School of Public Health, Sun Yat-sen University, Guangzhou 510080, China

**Keywords:** dietary calcium intake, blood pressure, hypertension, pre-hypertension, plant-based diet

## Abstract

This study aimed to assess the association of habitually low dietary calcium intake with blood pressure or hypertensive risk using data from the China Health and Nutrition Survey (CHNS) in 2009. We included 6298 participants (2890 men and 3408 women) aged 18 years or older in this analysis. Food intakes were measured by 3-day 24-h individual recalls combined with a weighing and measuring of household food inventory. The participants were divided into normotensive, pre-hypertensive and hypertensive groups according to their mean blood pressure of three repeated measurements. Six intake levels were decided by percentiles of gender-specific dietary calcium intakes (P0–10, P10–30, P30–50, P50–70, P70–90, and P90–100). Average dietary calcium intakes were 405 mg/day for men and 370 mg/day for women, 80% and 84% of which were derived from plant-based food in men and women, respectively. Multiple linear regression analyses showed that dietary calcium intakes were not related with blood pressure in both genders (all *P* > 0.05). Logistic regression analyses showed a lower risk of pre-hypertension with higher dietary calcium intakes in women (all *P*_for trend_ < 0.001), but not in men; no association between dietary calcium intake and hypertensive risk was found in both genders (all *P*_for trend_ > 0.05). This study suggests that there are no conclusive associations of habitually low dietary calcium intake with blood pressure or hypertensive risk in Chinese individuals consuming predominantly plant-based diets.

## 1. Introduction

A rising prevalence of hypertension worldwide has been well documented in the past two decades [1]. Studies have shown that dietary behaviors may influence the development of hypertension [2,3,4]. Evidence from a number of epidemiological studies indicated that low dietary calcium constituted a significant risk factor for primary hypertension and higher calcium intake helped to reduce blood pressure [5,6,7,8,9,10], while other studies reported no associations [11,12,13,14] or even a positive association [15]. Chinese individuals have traditionally consumed predominantly plant-based diets, characterized by limited dairy foods and rich in vegetables and soy products [16]. This dietary pattern provided about 400 mg/day of calcium [17,18], which was about half of the intake of Americans and Europeans [6,7,8,19], and was also lower than that in South Korea and Japan [5,20]. Such a low dietary calcium intake was reported to be related to increasing blood pressure in western individuals, who usually consumed 700–800 mg/day or more dietary calcium [14,21]. However, an improved understanding of the association between dietary calcium and hypertension is needed in the scope of habitually low dietary calcium intakes.

Fewer studies have documented this relationship among Chinese adults. Previous cross-sectional surveys conducted in Guangxi province, Tianjin province, and rural Henan province, China, found inverse associations between dietary calcium and blood pressure or hypertension among populations in both Han and minority nationalities [22,23,24,25]. While in contrast, investigations undertaken in Beijing and Guangdong province and another investigation in Tianjin did not find any association [26,27]. Given that the conflicting results may be due to different survey methods, and existing studies are limited by the relatively small sample size or restricted to specific groups; we therefore, investigated the association of low dietary calcium intake with blood pressure and hypertension using data from the China Health and Nutrition Survey (CHNS) [28], an ongoing, open cohort study established in 1989 and conducted in nine provinces of China. In this study, we only included participants taking part in the CHNS 2009, considering that there was no significant secular change in dietary calcium intake of Chinese adults up to 2009 [17,18], and serum biomarkers associated with blood pressure, such as blood glucose and blood lipids, were only measured in 2009.

## 2. Methods

### 2.1. Study Population

Cross-sectional data from the CHNS 2009 were used. This survey was conducted in nine provinces in China, with a multistage, randomized cluster design. The design and methods of this survey have been described in detail elsewhere [28]. All of the participants provided information on demographic and socioeconomic characteristics, lifestyles, usual diet, and physical examinations. Each participant recruited into the study signed an informed consent. The study protocol was approved by the Institutional Review Board at the China Center for Disease Control and Prevention (CDC) and the University of North Carolina at Chapel Hill.

In total, 8641 individuals aged 18 years and older from 218 communities were surveyed in the CHNS 2009. All participants were asked to finish a structured questionnaire, a dietary survey, physical examination and blood draw. The exclusion criteria include: those who had self-reported hypertension, diabetes, dyslipidemia; those who were currently taking antihypertensive, antidiabetic drugs; those who were diagnosed with severe diseases (including cancer, myocardial infarction, heart failure, stroke, thyroid dysfunction, inflammatory response, blindness, physical disability, etc.), and those who were pregnant and nursing. After excluding those with missing or implausible values, i.e., systolic blood pressure (SBP) <40 mmHg or >300 mmHg, diastolic blood pressure (DBP) <30 mmHg or >200 mmHg, energy intakes >5000 kcal/day or <500 kcal/day, BMI <14 kg/m^2^ or >45kg/m^2^, and nutrient intakes (mainly dietary calcium, carbohydrate, total fat, protein, fiber, sodium, potassium, magnesium and phosphorus intake) out of the 99% range of the population, 6298 adults were included in the current analysis, among which 2890 were men and 3408 were women.

#### 2.1.1. Hypertension Identification

Blood pressure was measured three times every 1–2 minutes following a standard procedure with a regularly calibrated mercury sphygmomanometer with appropriate-sized cuffs in the morning by trained physicians. Participants were required to avoid active exercises, smoking and talks, emptying the bladder within one hour, and keeping in a mental relaxed state for at least 10 minutes before measurements. In addition, they were asked to avoid taking foods, hot water or drugs that may affect blood pressure within 30 minutes. They could choose a sitting or horizontal position, but were asked to keep their arms and hearts at the same level. The average of the three readings was chosen as the corresponding BP values to reduce the effect of measurement errors. Blood pressure was recorded with mmHg as the unit. Subjects were classified into normotensive, pre-hypertensive, and hypertensive groups according to the classification of hypertension in Joint National Committee Report (JNC-Ш) [29] and 2010 Chinese guidelines for the management of hypertension [30]. Hypertensive cases were identified if a participant had a mean SBP/DBP ≥140/90 mmHg. Pre-hypertensive was defined by a mean SBP between 120 and 139 mmHg or a mean DBP between 80 and 89 mmHg. Only participants with both SBP <120 mmHg and mean DBP <80 mmHg were regarded as normotensive individuals.

#### 2.1.2. Dietary Intake Measures

The individual diet was assessed by three consecutive day 24-h recalls including one weekend day and two weekdays, in combination with the consumption of condiments (mainly cooking oil, salt, sauces, vinegar, etc.) recorded using household-based accounting method, adjusting for personal energy intake afterwards [28]. Energy and nutrient intakes were estimated by multiplying the energy and nutrient content of a standard portion size of 100 g by the consumption of each food item. Nutrient contents of food were obtained from the China Food Composition Tables [31,32]. Daily nutrient intakes were adjusted for total energy intake with the residual method [33]. 

#### 2.1.3. Anthropometric and Biomarker Measurements

Body weight and height were measured after subjects had their shoes, hats and heavy out garments removed. All of them took a stand-up posture, with their chest and abdomen held up, shoulders flat, arms drooped naturally, eyes flat front and head upright. Body weight was measured to the nearest 0.1 kg and height was measured to the nearest 0.1 cm. Body mass index (BMI) was calculated by weight in kg by the square of height in meter [BMI (kg/m^2^) = weight (kg)/height(m)^2^]. Waist circumference was measured under a standing posture with hands naturally sagging, then using a tape around the waist below the lowest rib margin and reading the measurement at minimum respiration. 

Fasting blood samples were collected from antecubital veins for biochemical tests. All serum and plasma samples were stored when internal quality control met the acceptable criteria, and were tested at a national central laboratory in Beijing (medical laboratory accreditation certificate ISO 15189:2007). Fasting blood-glucose (FBG) and blood lipid profiles, including total cholesterol (TC), triglycerides (TG), high-density lipoprotein cholesterol (HDL-C) and low-density lipoprotein cholesterol (LDL-C) were included in this study, all with mmol/L as units. 

#### 2.1.4. Other Demographic and Lifestyle Covariates

Demographic and lifestyle variables including age, residence (urban and rural areas), nationality (Han and non-Han Chinese), level of education [primary (primary school or below), middle (middle and high school or technical secondary school), and high (college or above)], smoking status (current smoker, former smoker and non-smoker), alcohol drinking and physical activity were obtained through questionnaire interview. The average daily alcohol consumption was calculated based on frequency and amount of alcohol consumed by the drinkers in the past year. Physical activity levels(PAL) was quantified into multiples of basal metabolic rate (BMR) on the basis of the Chinese Dietary Reference Intakes (2000 edition) [34]: in men, 1.3 × BMR or less for very light physical activity, 1.6 × BMR for light physical activity, 1.7 × BMR for moderate physical activity, 2.1 × BMR for heavy physical activity, and 2.4 × BMR or more for very heavy physical activity; in women, 1.3 × BMR or less for very light physical activity, 1.5 × BMR for light physical activity, 1.6 × BMR for moderate physical activity, 1.9 × BMR for heavy physical activity, and 2.2 × BMR or more for very heavy physical activity. In this survey, physical activities were also divided into five categories according to the type of professional activities, so we use multiple of BMR as an alternative of PAL. Urbanization index [35] was used to reflect demographic, economic, social, and infrastructural diversity at community level. Since calcium absorption is regulated by vitamin D, a prohormone mainly synthesized in the skin through exposure to sunlight [36], we used annual direct normal radiation (DNR) of each provincial capital obtained from the American National Aeronautics and Space Administration (NASA) Surface Meteorology and Solar Energy-Location as a surrogate for vitamin D status [37]. The latitude and longitude were adopted from Google earth Google Maps to locate the study sites [38]. 

### 2.2. Statistical Analysiss

Continuous variables were presented as means and standard deviations for normal distribution, medians and interquartile range for skewed distribution; and categorical variables were presented as numbers and proportions. Participants were divided into six intake levels (P0–10, P10–30, P30–50, P50–70, P70–90 and P90–100) according to the percentiles of gender-specific dietary calcium intake. Differences across dietary calcium intake levels were analyzed by using one-way ANOVA (normal distribution) or Kruskal–Wallis test (skewed distribution) for continuous variables with tests for linear trend, and by using *χ^2^* tests with linear-by-linear tests for categorical variables. Multiple linear regression models were used to investigate the association between dietary calcium intake and blood pressure. Demographic variables consisting of age, education level, ethnicity, residence, urbanization index were included in model 1. Total energy intake and BMI were included in each model for adjusting individual differences. Model 2 adjusted variables cited above in model 1 and further adjusted life behavior factors (including smoking status, alcohol consumption and PAL) [39]. Results from many studies indicated that there were various relationships among dietary nutrients and blood pressure or hypertension [3,40], and dietary mineral intake such as calcium, sodium, potassium, magnesium, and these may jointly affect blood pressure [5,41,42], so we further included dietary macronutrients, mineral nutrients, DNR and latitude as covariates in model 3. FBG and blood lipids (TG, HDL-C and LDL-C) as metabolic components of metabolic syndrome (MetS) [43] were further adjusted in model 4. Multinomial logistic regression analysis was used to examine the association between dietary calcium intake and risk of pre-hypertension and hypertension with the lowest percentile of dietary calcium intake (P0-P10) as reference. The adjusted variables in the models were the same with multivariable linear regression models above. Waist circumference, dietary carbohydrate and phosphorus intake and total cholesterol were excluded for collinearity in the models before analysis, and there is no collinearity among all the variables in the final analysis.

Due to the significant gender differences in blood pressure and calcium intake, and the gender differences that were observed in previous studies for assessing the association of calcium and blood pressure or the risk of hypertension [44], men and women were analyzed separately. The statistical analyses were performed by SPSS version 22.0 software (SPSS Inc., Chicago, IL, USA) for WINDOWS. A two-tailed *P* < 0.05 was considered as the significant level for all analyses. 

## 3. Results

The baseline characteristics of men and women are, respectively, shown in Table 1. The average SBP/DBP were 123/81 mmHg in men and 119/77 mmHg in women. Higher proportions were observed in men than women of hypertension and prehypertension (25.3% vs. 19.1%; 23.4 vs.17.1%, respectively). The mean age was 48.5 years in men, and 48.3 years in women. Average dietary calcium intakes were 405 mg/d in men and 370 mg/d in women, about 80% and 84% of which were derived from plant food, respectively. Men and women (averagely) consumed 193 mg/day and 171 mg/day of dietary calcium intake in the lowest 10% percentile (P0–P10), and 767 mg/day and 725 mg/day in the highest 10% percentile (P90–P100), respectively. There was no significant difference in SBP and DBP throughout the six calcium intake levels in both men and women. The distribution of normotensive, pre-hypertensive and hypertensive participants was significantly different across the calcium intake levels in women, but not in men. 

The associations between dietary calcium intake and blood pressure for men and women are shown in Table 2. After adjusting for age, neither SBP nor DBP showed significant correlation with dietary calcium intake in men or women (all *P* > 0.05, Table 2). Furthermore, after adjusting for other covariates, there was no significant correlation between dietary calcium intake and SBP or DBP of both genders in all multiple linear models (all *P* > 0.05).

The associations between dietary calcium intake and risk of hypertension and pre-hypertension are presented in Table 3. Compared with the lowest percentile of calcium intake (P0–P10), women whose dietary calcium intake above P10 had significantly lower risk of pre-hypertension, and the OR (95%CI) for percentile ranges of P10–30, P30–50, P50–70, P70–90 and P90–100 were 0.62(0.44, 0.89), 0.68(0.48, 0.98) 0.67(0.46, 0.97), 0.60(0.40, 0.89) and 0.32(0.19, 0.55) in model 3 (*P*_for trend_ = 0.001); and 0.65(0.45, 0.93), 0.71(0.50, 1.03), 0.71(0.48, 1.03), 0.63(0.43, 0.94), 0.33(0.20, 0.56) in model 4 (*P*_for trend_ = 0.001). However, we did not find similar associations in men (all *P*_for trend_ > 0.05). No associations were observed between dietary calcium intakes and the risk of hypertension in men or women (all *P*_for trend_ > 0.05).

## 4. Discussion

This cross-sectional study showed that there was no significant or consistent association between low dietary calcium intake and blood pressure in Chinese adults with predominantly plant-based diets, whose mean dietary calcium intake was below 400 mg/day. Although such a low intake of dietary calcium was reported as a risk factor for hypertension in western populations [44], our findings did not support a clear relationship between dietary calcium intake and hypertension in the scope of habitually low dietary calcium intakes, except that a lower risk of pre-hypertension was observed to be associated with higher dietary calcium intake in women. 

Inconsistent associations were found between dietary calcium intake and hypertension in previous studies. Some studies reported that lower calcium intake was associated with blood pressure or hypertension [9,44,45,46,47,48,49]. Data from the National Health and Nutrition Examination (NHANES) was early used to explore the relation of nutrient intake to blood pressure in American adults. The study of Mccarron, et al. using the NHANES data found that lower intake of calcium was the most consistent factor in hypertensive subjects (hypertensive vs. normotensive individuals: 673 mg/day vs. 728 mg/day), and it was associated with higher mean SBP and a higher risk of hypertension [9]. Subsequent studies using the data from the NHANES 2001–2010 (860 mg/day dietary calcium in men and 773 mg/day in women) also indicated a significantly inverse association between calcium intake and hypertensive risk [8]. Additionally, studies conducted in Europe and Eastern Asia also revealed an inverse association of higher blood pressure or hypertensive risk with low dietary calcium at various average or median intakes, such as in the Mediterranean (987.2 mg/day for normotensive, 953.8 mg/day for non-medicated hypertensive subjects) [7], Poland (816 mg/day) [46], France (1109 mg/day in men and 980 mg/day in women) [47], Belgium (841 mg/day in men) [50], Taiwan of China (658 mg/day in men and 562 mg/day in women) [51], Japan (528–639 mg/day in women) [20] and South Korea (605.8 mg/day for 30–49 years) [5]. However, other studies found no association between calcium intake and blood pressure in American white (702 mg/day in NHANES І, 699 mg/day in NHANES II) [14], Alaska Native (1202 mg/day in men; 974 mg/day in women) [52], Spanish (about 1200 mg/day) [53], Japanese (449–669 mg/day for 40–69 years) [54] and South Korean (KNHANES III, 536.8 mg/day for normotensive and 525.9 mg/day for hypertensive) populations [41]. The average dietary calcium intake (405 mg/day in men and 370 mg/day in women) in our study was much lower than that of westerners, even lower than that of Japanese and South Koreans. We have not found a report on the association between blood pressure and dietary calcium consumption at such low intakes. One possible explanation for the current study is that although calcium intake of Chinese is low, calcium concentration in the body has reached a state of equilibrium, and the current level of intake can already meet the regulation of blood pressure. Studies have shown that calcium absorption is negatively correlated with calcium intake [55,56]. The human can adapt to a habitually low calcium diet by increasing calcium absorption in the gastrointestinal tract and by reducing the calcium excretion in the kidney [57,58]. In addition, dietary calcium consumed by the Chinese sourced from eggs, fish, shrimps and milk were less than 5%, while from vegetables, soy products and cereals were about 35%, 20% and 17%, respectively [17,18]. Whereas nearly one third of dietary calcium in Japanese [59] and Korean [60] and more than 80% of dietary calcium in westerners [61,62] were derived from dairy and meat products. Although dairy is thought to be the best dietary source for calcium, calcium balance studies indicated that the absorption and bioavailability of calcium sourced from some vegetables and soy products could be as good as or even better than that from dairy foods [63,64,65]. Moreover, besides calcium intake and sources, calcium bioavailability was also affected by other components in mixed diets except phytic acid, and hormones such as phosphorus, vitamin D, parathyroid hormone, and calcitonin [57,66,67].

A study from the United States suggested that a calcium intake of less than 700–800 mg/day was associated with a higher risk of hypertension [21]. In the present study, we divided present dietary calcium into six intake levels to make a comparison with western populations. The average dietary calcium intakes of Chinese individuals in the top 10 percentile level (P90–P100) was 767mg/day in men and 725mg/day in women, which were comparable to the western population in the scope of 700–800 mg/day. However, no protective effects were found on hypertension at such high intake levels of dietary calcium. Blood pressure is regulated by circulating calcium concentration, which varies within a relatively small range, despite a wide range of dietary calcium intakes among individuals. The human body can adapt to different dietary calcium intakes and maintain calcium equilibrium by regulating calcium absorption and output through urine and feces [57,58,68]. The relation between dietary calcium and blood pressure is an outcome of long-term adaptation, and habitual intake of dietary calcium for an individual usually varies within a relatively stable range [17,18]. Short-term intervention of calcium supplements with daily dosage at hundreds of milligrams was reported to be effective in reducing blood pressure [69], which was different from habitual dietary calcium from food source. It is, thus, not reasonable to extend the conclusion of calcium supplements to dietary calcium, and the correlation between dietary calcium and hypertensive risk may be weak by contrast, if it does exist. Therefore, taking all the evidence together, it is still an open question whether higher dietary calcium intake has beneficial effects on blood pressure and hypertension or not, and more in-depth research is needed.

Strengths of our study are the relatively large sample size and good representativeness with participants recruited from nine provinces in China. In this study, pre-hypertension and hypertension were analyzed as outcome variables, and biochemical indicators were also included as confounding factors. Beyond all of the above, we divided calcium intake into six intake levels to make the highest level close to average dietary calcium intakes in western countries, so as to make better comparison with the findings from Europe and the United States. 

This study also has several limitations. First, this is a cross-sectional investigation, longitudinal changes of blood pressure of this population cannot be observed in this analysis. Second, some confounding factors such as family history of hypertension and other possible covariates were not included. Third, there is a lack of information on calcium supplementation in this survey, although the proportion of Chinese individuals using dietary supplements was quite low [70]. Fourth, this survey is not nationally representative. Fifth, dietary calcium consumed by the Chinese was mainly derived from plant food, which is always rich in phytochemicals, such as flavonoid, which was reported to influence blood pressure as well [71,72]. However, information on content of phytochemicals in food was unavailable currently in the China Food Composition Tables, we could not gain an insight into the interaction effects between phytochemicals and calcium and blood pressure.

## 5. Conclusions

In conclusion, this study based on data from the CHNS 2009, suggested no conclusive associations between habitually low dietary calcium intake and blood pressure or hypertensive risk in Chinese adults with a predominantly plant-based diet containing a relatively low dietary calcium intake of 400 mg/day on average.

## Figures and Tables

**Table 1:** Characteristics and nutrient intake by different percentiles of dietary calcium intake in men and women.

**Variable ^1^**	**Percentiles of Dietary Calcium Intakes Per Day**							***P*_for trend_**
	**Total**	**P0–10**	**P10–30**	**P30–50**	**P50–70**	**P70–90**	**P90–100**	
**Men**	***n* = 2890**	***n* = 289**	***n* = 578**	***n* = 578**	***n* = 578**	***n* = 578**	***n* = 289**	
Age (years)	48.5 ± 15.0	50.2 ± 15.9	48.6 ± 15.4	47.3 ± 14.3	48.9 ± 15.1	47.8 ± 14.9	49.3 ± 14.3	0.446
SBP (mmHg)	122.9 ± 15.3	123.1 ± 16.9	122.7 ± 15.2	122.6 ± 13.8	123.6 ± 15	122.5 ± 15	123.4 ± 17.5	0.800
DBP (mmHg)	80.8 ± 10.2	80.4 ± 11.4	80.3 ± 10.2	80.9 ± 9.8	81.2 ± 10.2	80.7 ± 9.7	81.1 ± 10.9	0.293
Blood pressure level (%)								0.829
Normotensive	51.2	52.9	52.6	51.7	45.5	52.8	54.3	
Pre-hypertensive	25.3	20.1	25.3	25.4	30.4	24.7	21.5	
Hypertensive	23.4	27.0	22.1	22.8	24.0	22.5	24.2	
Urban (%)	30.5	17.6	18.9	28.9	33.7	40	44.3	<0.001
Urbanization Index								<0.001
Median	62.0	57.6	60.3	60.3	64.2	67.5	71.4	
25th, 75th	50.6, 84.2	49.6,73.9	47.9, 81.6	49.6, 83.7	50.9, 84.0	51.1, 87.8	51.3, 89.2	
Education level								0.004
Primary (%)	32.8	35.6	37.9	28.7	33.4	31.0	30.1	
Middle (%)	60.8	60.2	57.6	65.1	59.7	60.7	61.9	
High (%)	6.4	4.2	4.5	6.2	6.9	8.3	8.0	
Ethnicity								
Han nationality (%)	88.1	82.7	88.2	89.1	90.3	89.2	87.2	0.052
Smoking Status								0.230
Non (%)	20.6	16.6	20.8	22.3	21.6	20.2	19.7	
Former (%)	5.3	3.8	5.0	3.8	6.1	6.7	5.9	
Current (%)	74.1	79.6	74.2	73.9	72.3	73	74.4	
Alcohol consumption (g/day)								0.351
Median	4.3	3.2	4.3	6.4	4.3	6.4	6.3	
25th, 75th	0.0, 24.6	0.0, 24.3	0.0, 24.6	0.0, 25.7	0.0, 22.5	0.0, 21.4	0.0, 27.6	
PAL (×BMR)	1.8 ± 0.3	1.8 ± 0.3	1.8 ± 0.3	1.8 ± 0.3	1.8 ± 0.3	1.7 ± 0.3	1.7 ± 0.3	<0.001
BMI (kg/m^2^)	23.1 ± 3.4	22.4 ± 3	23.0 ± 3.3	23.2 ± 3.3	23.2 ± 3.3	23.1 ± 3.5	23.4 ± 3.6	0.001
WC (cm)	83.4 ± 9.9	81.8 ± 8.8	83 ± 10	83.7 ± 10.1	83.8 ± 9.9	83.4 ± 10	84.7 ± 10.5	0.011
Biomarkers								
FBG (mol/L)	5.3 ± 1.3	5.4 ± 1.5	5.3 ± 1.3	5.3 ± 1.2	5.3 ± 1.4	5.3 ± 1.4	5.3 ± 1.2	0.605
TC (mmol/L)	4.8 ± 1.0	4.8 ± 1.0	4.7 ± 1.0	4.7 ± 1.0	4.8 ± 1.0	4.8 ± 1.0	4.9 ± 1.0	0.164
TG (mmol/L)	1.7 ± 1.5	1.7 ± 1.4	1.7 ± 1.6	1.6 ± 1.1	1.8 ± 1.9	1.7 ± 1.4	1.8 ± 1.5	0.172
HDL-C (mmol/L)	1.4 ± 0.5	1.4 ± 0.5	1.4 ± 0.4	1.4 ± 0.5	1.4 ± 0.6	1.4 ± 0.4	1.4 ± 0.6	0.970
LDL-C (mmol/L)	2.9 ± 1.08	2.9 ± 0.9	2.9 ± 1.1	2.9 ± 0.9	2.9 ± 0.9	2.9 ± 0.9	3 ± 0.9	0.677
Daily nutrient intakes								
Total energy (kcal/day)	2377 ± 653	2326 ± 657	2413 ± 625	2375 ± 646	2371 ± 641	2381 ± 692	2358 ± 661	0.844
CHO (g/day)	351 ± 67	352 ± 72	363 ± 65	360 ± 65	351 ± 64	339 ± 69	335 ± 67	<0.001
Fat (g/day)	85 ± 27	86 ± 29	83 ± 28	83 ± 26	85 ± 26	87 ± 28	86 ± 27	0.166
Protein (g/day)	74 ± 16	63 ± 13	68 ± 14	71 ± 13	75 ± 13	80 ± 16	87 ± 17	<0.001
Na (mg/day)	5197 ± 3299	4334 ± 2342	5016 ± 3099	5200 ± 2943	5128 ± 2663	5398 ± 3314	6154 ± 5393	<0.001
K (mg/day)	1800 ± 477	1366 ± 342	1606 ± 339	1739 ± 385	1828 ± 369	2018 ± 464	2257 ± 599	<0.001
Mg (mg/day)	323 ± 76	254 ± 58	298 ± 61	315 ± 62	326 ± 61	354 ± 77	395 ± 82	<0.001
P (mg/day)	1076 ± 276	913 ± 175	995 ± 169	1043 ± 168	1087 ± 161	1163 ± 209	1270 ± 217	<0.001
Ca (mg/day)	405 ± 166	193 ± 32	270 ± 21	336 ± 19	410 ± 24	527 ± 49	767 ± 126	<0.001
Ca from plant food (mg/day)	323 ± 143	156 ± 36	226 ± 37	285 ± 39	342 ± 62	428 ± 100	589 ± 190	<0.001
Ca from animal food (mg/day)	61 ± 42	27 ± 19	29 ± 24	36 ± 29	49 ± 50	75 ± 84	144 ± 159	<0.001
Dietary fiber (g/day)	12.4 ± 6.0	9.6 ± 6.1	11.5 ± 5.6	12.2 ± 5.1	12.6 ± 5.7	13.7 ± 6.1	14.5 ± 7.3	<0.001
DNR (kWh/m^2^/day)	32.7 ± 6.9	30.7 ± 6.1	30.7 ± 6.1	32.9 ± 7.3	33.4 ± 7.3	32.7 ± 6.8	32.8 ± 6.7	<0.001
Latitude	3.6 ± 1.0	3.2 ± 0.9	3.6 ± 1.0	3.7 ± 1.0	3.7 ± 1.0	3.7 ± 1.1	3.8 ± 1.1	<0.001
**Women**	***n* = 3408**	***n* = 340**	***n* = 682**	***n* = 682**	***n* = 682**	***n* = 682**	***n* = 340**	
Age (years)	48.3 ± 14.2	47.9 ± 15.8	48.4 ± 14.2	47.8 ± 14.3	48.2 ± 14.1	48.1 ± 13.9	49.6 ± 13.7	0.160
SBP (mmHg)	119.4 ± 16.8 ^2^	120.2 ± 17.6	119.3 ± 16.6	119.5 ± 16.5	119.6 ± 16.8	118.5 ± 16.9	119.8 ± 17.2	0.512
DBP (mmHg)	77.4 ± 10.2 ^2^	77.6 ± 11	77 ± 10.2	77.6 ± 9.9	77.5 ± 10.2	77 ± 9.8	77.8 ± 10.3	0.890
Blood pressure level (%)								0.024
Normotensive	63.8 ^2^	57.9	65.4	63.0	62.5	66.0	66.2	
Pre-hypertensive	19.1 ^2^	21.8	19.5	20.4	20.4	18.6	11.8	
Hypertensive	17.1 ^2^	20.3	15.1	16.6	17.2	15.4	22.1	
Urban (%)	31.0	15.6	19.9	27.9	33.4	39.7	52.6	<0.001
Urbanization Index								<0.001
Median	63.1	58.9	58.7	60.7	66.7	70.6	82.1	
25th, 75th	51.1, 85.1	49.7, 76.0	47.6, 80.0	49.7, 84.2	51.3, 85.0	53.3, 89.5	54.3, 90.6	
Education level								<0.001
Primary (%)	47.4 ^2^	50	53.4	47.4	46	45.2	40.6	
Middle (%)	48.5 ^2^	48.2	44	49.6	49.1	48.7	54.1	
High (%)	4.0 ^2^	1.8	2.6	3.1	4.8	6.2	5.3	
Ethnicity								
Han nationality (%)	87.8	83.7	88.3	88.4	88.7	88.4	90	0.020
Smoking Status								0.195
Never (%)	93.4 ^2^	92.1	93.1	92.5	94.3	94.4	93.5	
Former (%)	0.4 ^2^	0.3	0.1	0.7	0.1	0.4	0.3	
Current (%)	6.2	7.6	6.7	6.7	5.6	5.1	6.2	
Alcohol consumption (g/day)								0.726
Median	0.0 ^2^	0.0	0.0	0.0	0.0	0.0	0.0	
25th, 75th	0.0,0.0	0.0, 0.0	0.0, 0.0	0.0, 0.0	0.0, 0.0	0.0, 0.0	0.0, 0.0	
PAL (×BMR)	1.6 ± 0.2 ^2^	1.6 ± 0.2	1.6 ± 0.2	1.6 ± 0.2	1.6 ± 0.2	1.6 ± 0.2	1.5 ± 0.2	<0.001
BMI (kg/m2)	23.1 ± 3.4	23.1 ± 3.6	23 ± 3.4	23.1 ± 3.4	23.1 ± 3.5	23.2 ± 3.3	23.2 ± 3.3	0.553
WC (cm)	80.2 ± 9.9	80.6 ± 10.2	79.9 ± 9.7	80.1 ± 9.9	79.9 ± 10	80.8 ± 10.1	80.4 ± 9.3	0.437
Biomarkers								
FBG (mol/L)	5.2 ± 1.0 ^2^	5.3 ± 1.0	5.1 ± 1.0	5.2 ± 1.0	5.2 ± 1.0	5.2 ± 0.9	5.2 ± 0.9	0.706
TC (mmol/L)	4.8 ± 1.0	4.9 ± 1.0	4.8 ± 1.0	4.8 ± 1.0	4.8 ± 1.0	4.8 ± 0.9	4.8 ± 1.0	0.834
TG (mmol/L)	1.5 ± 1.1 ^2^	1.6 ± 1.3	1.4 ± 1.1	1.5 ± 1.0	1.4 ± 0.9	1.4 ± 1.0	1.6 ± 1.7	0.678
HDL-C (mmol/L)	1.5 ± 0.4 ^2^	1.5 ± 0.6	1.5 ± 0.4	1.5 ± 0.4	1.5 ± 0.4	1.5 ± 0.4	1.5 ± 0.3	0.738
LDL-C (mmol/L)	3.0 ± 0.9 ^2^	3.0 ± 1.1	2.9 ± 0.9	2.9 ± 0.9	2.9 ± 0.9	3 ± 0.9	3.0 ± 1.0	0.669
Daily nutrient intakes								
Total energy (kcal/day)	2009 ± 580 ^2^	1934 ± 573	2040 ± 558	2034 ± 596	2010 ± 600	2031 ± 579	1929 ± 544	0.748
CHO (g/day)	302.9 ± 55.5 ^2^	308 ± 56	312 ± 56	308 ± 55	304 ± 54	293 ± 54	287 ± 56	<0.001
Fat (g/day)	74 ± 24 ^2^	74 ± 26	72 ± 25	73 ± 24	73 ± 24	76 ± 23	75 ± 24	0.070
Protein (g/day)	64 ± 14 ^2^	54 ± 11	59 ± 12	61 ± 11	65 ± 12	69 ± 13	77 ± 15	<0.001
Na (mg/day)	4538 ± 2740 ^2^	3900 ± 1977	4449 ± 2643	4549 ± 2720	4511 ± 2582	4793 ± 2929	4878 ± 3368	<0.001
K (mg/day)	1624 ± 452 ^2^	1232 ± 336	1443 ± 324	1556 ± 375	1664 ± 373	1808 ± 434	2064 ± 540	<0.001
Mg (mg/day)	285 ± 68 ^2^	223 ± 55	260 ± 54	278 ± 58	292 ± 59	309 ± 65	350 ± 69	<0.001
P (mg/day)	940 ± 186 ^2^	795 ± 160	866 ± 152	905 ± 144	954 ± 150	1015 ± 193	1129 ± 176	<0.001
Ca (mg/day)	370 ± 160 ^2^	171 ± 26	242 ± 19	303 ± 18	373 ± 23	486 ± 48	725 ± 126	<0.001
Ca from plant food (mg/day)	325 ± 145	140 ± 30	203 ± 33	260 ± 38	316 ± 55	396 ± 94	563 ± 189	<0.001
Ca from animal food (mg/day)	60 ± 44	23 ± 18	27 ± 23	31 ± 28	43 ± 48	74 ± 87	141 ± 137	<0.001
Dietary fiber (g/day)	11.5 ± 5.6 ^2^	9.0 ± 6.1	10.7 ± 4.7	11.1 ± 4.9	11.6 ± 4.8	12.8 ± 6.7	13.3 ± 5.8	<0.001
DNR (kWh/m^2^/day)	33.0 ± 6.8	31.4 ± 6.6	33.5 ± 7.2	33.6 ± 7.4	33.3 ± 6.7	32.3 ± 6.1	33.5 ± 6.3	<0.001
latitude	3.7 ± 1.0	3.3 ± 1.0	3.7 ± 1.0	3.8 ± 1.0	3.8 ± 1.1	3.7 ± 1.1	3.8 ± 1.1	<0.001

SBP, systolic blood pressure; DBP, diastolic blood pressure; PAL, physical activity level; BMR, basal metabolic rate; BMI, body mass index; WC: waist circumference; FBG, fasting blood glucose; TC, total cholesterol; TG, triacylglycerol; HDL-C, high density lipoprotein cholesterol; LDL-C, low density lipoprotein cholesterol; CHO, carbohydrates; DNR, annual average direct normal radiation. **^1^** Data were shown with mean ± SD, percentage (%) or median (25–75% interquartile range); ^2^ Compared with men, *P* < 0.05.

**Table 2:** Multivariable coefficients (β and SE) of systolic and diastolic blood pressure with dietary calcium intake of men and women.

	SBP			DBP		
	β	SE	*P*	β	SE	*P*
**Men (*n* = 2890)**						
		*r*^2^ = 0.117			*r*^2^ = 0.019	
Age adjusted model	0.001	0.002	0.672	0.001	0.001	0.398
		*r*^2^ = 0.171			*r*^2^ = 0.102	
Multivariate model 1	0.000	0.002	0.775	0.000	0.001	0.832
		*r*^2^ = 0.174			*r*^2^ = 0.108	
Multivariate model 2	0.000	0.002	0.837	0.000	0.001	0.871
		*r*^2^ = 0.184			*r*^2^ = 0.137	
Multivariate model 3	−0.001	0.002	0.366	0.001	0.001	0.757
		*r*^2^ = 0.182			*r*^2^ = 0.145	
Multivariate model 4	−0.002	0.002	0.267	0.000	0.001	0.932
**Women (*n* = 3408)**						
		*r*^2^ = 0.199			*r*^2^ = 0.072	
Age adjusted model	−0.001	0.002	0.557	0.000	0.001	0.762
		*r*^2^ = 0.258			*r*^2^ = 0.155	
Multivariate model 1	−0.001	0.002	0.580	0.001	0.001	0.579
		*r*^2^ = 0.258			*r*^2^ = 0.153	
Multivariate model 2	−0.001	0.002	0.584	0.001	0.001	0.593
		*r*^2^ = 0.267			*r*^2^ = 0.184	
Multivariate model 3	0.000	0.002	0.774	0.001	0.001	0.393
		*r*^2^ = 0.272			*r*^2^ = 0.192	
Multivariate model 4	0.000	0.002	0.773	0.001	0.001	0.364

SBP, systolic blood pressure; DBP, diastolic blood pressure; SE, standard error. Model 1 was adjusted for age, education level, ethnicity, residence, urbanization index, BMI and total energy intake; Model 2 was further adjusted for smoking status, alcohol consumption, physical activity level (PAL); Model 3 was further adjusted for total fat intake, protein intake, dietary fiber intake, annual average direct normal radiation (DNR), latitude, dietary sodium, potassium and magnesium intake; Model 4 was further adjusted for fasting blood glucose (FBG), triacylglycerol (TG), high-density cholesterol (HDL-C), low-density lipoprotein cholesterol (LDL-C).

**Table 3:** Odds ratio (95% confidence interval) for pre-hypertension and hypertension with dietary calcium intakes of men and women.

	Percentiles of Dietary Calcium Intake Per Day						*P*_for trend_
	P0–10	P10–30	P30–50	P50–70	P70–90	P90–100	
Men (*n* = 2890)	*n* = 289	*n* = 578	*n* = 578	*n* = 578	*n* = 578	*n* = 289	
**Pre-hypertension**							
No. ^1^	58	146	147	176	143	62	
Age adjusted model	ref	1.29(0.90, 1.85)	1.34(0.93, 1.92)	1.79(1.25, 2.57)	1.27(0.88, 1.82)	1.05(0.69, 1.60)	0.324
Multivariate model 1	ref	1.19(0.82,1.71)	1.18(0.81,1.70)	1.58(1.10,2.28)	1.12(0.77,1.62)	0.89(0.58,1.38)	0.854
Multivariate model 2	ref	1.20(0.83,1.73)	1.18(0.82,1.72)	1.60(1.11,2.31)	1.13(0.78,1.63)	0.89(0.58,1.38)	0.837
Multivariate model 3	ref	1.05(0.72, 1.54)	1.02(0.69, 1.50)	1.43(0.97, 2.12)	0.99(0.65, 1.51)	0.78(0.47, 1.30)	0.981
Multivariate model 4	ref	1.07(0.73, 1.57)	1.03(0.70, 1.52)	1.42(0.96, 2.11)	0.99(0.65, 1.50)	0.77(0.46, 1.28)	0.942
**Hypertension**							
No. ^2^	78	128	132	139	130	70	
Age adjusted model	ref	0.87(0.61, 1.24)	0.98(0.69, 1.40)	1.10(0.77, 1.57)	0.92(0.65, 1.31)	0.91(0.61, 1.36)	0.928
Multivariate model 1	ref	0.76(0.91,1.89)	0.85(0.59,1.22)	0.99(0.68,1.42)	0.83(0.58,1.20)	0.79(0.52,1.21)	0.609
Multivariate model 2	ref	0.77(0.53,1.11)	0.85(0.59,1.23)	1.00(0.69,1.45)	0.84(0.58,1.21)	0.78(0.51,1.20)	0.605
Multivariate model 3	ref	0.68(0.47, 0.99)	0.75(0.51, 1.10)	0.91(0.61, 1.35)	0.75(0.49, 1.15)	0.70(0.42, 1.17)	0.499
Multivariate model 4	ref	0.69(0.47, 1.01)	0.75(0.51,1.11)	0.90(0.61, 1.34)	0.75(0.49, 1.14)	0.68(0.41, 1.14)	0.415
**Women (*n* = 3408)**	***n* = 340**	***n* = 682**	***n* = 682**	***n* = 682**	***n* = 682**	***n* = 340**	
**Pre-hypertension**							
No. ^1^	74	133	139	139	127	40	
Age adjusted model	ref	0.76 (0.54, 1.06)	0.84(0.60, 1.18)	0.84(0.60, 1.17)	0.72(0.51, 1.01)	0.44(0.28, 0.68)	0.004
Multivariate model 1	ref	0.75(0.53,1.06)	0.84(0.60,1.19)	0.84(0.60,1.18)	0.72(0.51,1.02)	0.44(0.28,0.69)	0.001
Multivariate model 2	ref	0.75(0.53,1.06)	0.84(0.72,1.69)	0.84(0.60,1.18)	0.73(0.51,1.03)	0.44(0.28,0.69)	0.001
Multivariate model 3	ref	0.62(0.44, 0.89)	0.68(0.48, 0.98)	0.67(0.46, 0.97)	0.60(0.40, 0.89)	0.32(0.19, 0.55)	0.001
Multivariate model 4	ref	0.65(0.45, 0.93)	0.71(0.50, 1.03)	0.71(0.48, 1.03)	0.63(0.43, 0.94)	0.33(0.20, 0.56)	0.001
**Hypertension**							
No. ^2^	69	103	113	117	105	75	
Age adjusted model	ref	0.62(0.43, 0.91)	0.75(0.52, 1.09)	0.77(0.53, 1.11)	0.64(0.44, 0.93)	0.87(0.58, 1.30)	0.935
Multivariate model 1	ref	0.63(0.43,0.93)	0.77(0.52,1.13)	0.79(0.54,1.16)	0.66(0.45,0.97)	0.95(0.62,1.46)	0.618
Multivariate model 2	ref	0.63(0.43,0.92)	0.77(0.52,1.12)	0.79(0.54,1.16)	0.65(0.44,0.96)	0.94(0.61,1.45)	0.627
Multivariate model 3	ref	0.59(0.40, 0.88)	0.75(0.50, 1.13)	0.81(0.53, 1.23)	0.73(0.47, 1.13)	1.15(0.68, 1.94)	0.079
Multivariate model 4	ref	0.61(0.41, 0.91)	0.78(0.52, 1.17)	0.86(0.56, 1.30)	0.77(0.49, 1.19)	1.18(0.70, 2.00)	0.067

Model 1 was adjusted for age, education level, ethnicity, residence, urbanization index, BMI and total energy intake; Model 2 was further adjusted for smoking status, alcohol consumption, physical activity level (PAL); Model 3 was further adjusted for total fat intake, protein intake, dietary fiber intake, annual average direct normal radiation (DNR), latitude, dietary sodium, potassium and magnesium intake; Model 4 was further adjusted for fasting blood glucose (FBG), triacylglycerol (TG), high-density cholesterol (HDL-C), low-density lipoprotein cholesterol (LDL-C). 1 Number of pre-hypertensive subjects. 2 Number of hypertensive subjects.
